# Chromosomal Replication, Translocation and Recombination as Putative Events in the Diversification of Vertebrate *AQP8*-Type Genes

**DOI:** 10.3390/ijms27093937

**Published:** 2026-04-28

**Authors:** Roderick Nigel Finn, Joan Cerdà

**Affiliations:** 1 Department of Biological Sciences, University of Bergen, 5006 Bergen, Norway; 2 Institute of Marine Sciences, Spanish National Research Council (CSIC), 08003 Barcelona, Spain; jcerda@icm.csic.es; 3 Institute of Biotechnology and Biomedicine (IBB), Universitat Autònoma de Barcelona, Bellaterra, 08193 Cerdanyola del Vallès, Spain

**Keywords:** orthology, aquaporin, translocation, recombination, duplication, pseudogene

## Abstract

AQP8-type water channels are expressed superficially in the plasma membrane or intracellularly in the inner mitochondrial membrane, where they respectively function in osmohomeostasis or as peroxiporins to alleviate oxidative stress. To date only single-copy *AQP8* or *AQP16* genes are known in tetrapods and two binary gene clusters composed of *aqp8aa-aqp8ab* and *aqp8ba-aqp8bb* in teleost fishes. Here, using phylogenomic and synteny analyses, we revise this view and show that bony fish *aqp8*aa, *-ab*, *-ba* and *-bb* genes are non-canonical co-orthologs that independently arose at chromosomal breakpoints. Conversely, canonical orthologs of tetrapod *AQP8* are now detected in all vertebrate classes except hagfishes. In cartilaginous fishes, intact *aqp8* orthologs and linked pseudogenes exist in squalomorph sharks and only fractionated *aqp8*-like pseudogenes in galeomorph sharks. Some isolated *aqp8*-like exons are detected in batoid ray genomes, while no aqp8-type coding sequences are currently found in holocephalan genomes. In the actinopterygian (ray-finned fish) lineage, the canonical ortholog of tetrapod *AQP8* is estimated to have undergone gene translocation in their common ancestor ~400 million years ago but was subsequently inactivated or lost in many descendant lineages. In close temporal proximity to this gene translocation event, the actinopterygian *aqp8aa*-*aqp8ab* binary gene cluster was generated in the original syntenic locus, potentially as a result of meiotic recombination. Our data support a model of total chromosomal replication for the generation of *AQP16* genes and the teleost *aqp8ba-aqp8bb* gene cluster. We further uncover additional duplicates in Strepsirrhini primates that provide an eminent example of the stochastic nature of neofunctionalization. The present data thus suggest that that common ancestral genome duplications combined with lineage-level chromosomal translocation, recombination and replication events contributed to the diversification of vertebrate *AQP8*-type genes.

## 1. Introduction

Aquaporin-8 (AQP8)-type water channels were first identified in the mammalian testis and shown to have similar structural features to other members of the aquaporin superfamily (Ishibashi et al., 1997) [1]. They are assembled as tetramers, with each monomer composed of six transmembrane domains (TMD1-6) linked by three extracellular (A, C, E) and two intracellular (B, D) disordered loops together with intracellular amino- (NT) and carboxy- (CT) termini [2,3]. Channel permeability is considered to be primarily regulated by the aromatic-arginine (ar/R) selectivity filter composed of four residues located on TMD2, -5 and loop E together with two conserved asparagine–proline–alanine (NPA) motifs that interface within the central pore of each monomer to facilitate the transmembrane flux of a range of small uncharged molecules including water, urea, ammonia, hydrogen peroxide (H_2_O_2_), and in some cases glycerol [1,4,5,6,7,8,9,10,11,12,13,14,15]. In both mammals and fishes, AQP8-type channels may be expressed in the plasma membrane or intracellularly in the inner mitochondrial membrane. In the former case, the channels are important for the homeosmotic regulation of cells in the spinal cord, brain, trachea, salivary glands, muscle, pancreas, kidney, liver, gall bladder, small intestine and testis [8,9,16,17,18,19,20,21,22,23,24]. Based upon studies of AQP8-deficient mice, the absence of the channel has to date been implicated in pregnancy changes, including increased numbers of multi-oocyte follicles, mature follicles and embryos, as well as elevated levels of amniotic fluid and neonatal mass [25,26,27,28]. Conversely, when expressed in the inner mitochondrial membrane of hepatic and granulosa cells or spermatozoa they can function as peroxiporins, mitigating oxidative stress through mitochondrial detoxification [11,14,15,20,29,30,31]. In such cases, the channels have been implicated in the hepatic regulation of cholesterogenesis, granulosa cell autophagy, and spermatozoon swimming performance for fertility competence [14,30,32].

Since their discovery, AQP8-type channels are now considered to have deep orthologous roots within Eukaryota [33,34], although certain lineages, such as arthropods, may have lost them [35,36,37,38]. In vertebrates and other non-arthropod metazoans, AQP8-type coding sequences (CDS) are thus phylogenetically distinct in that they form a separate cluster to those of classical water channels (AQP0, -1, -2, -4, -5, -6, -14, -15), aquaglyceroporins (AQP3, -7, -9, 10, -13) and unorthodox channels (AQP11, -12) [33,34]. The vertebrate AQP8-type branch of channels has nevertheless been shown to be composed of several subclades including single copy *AQP8* and *AQP16* genes in tetrapods and tetraparalogous *aqp8aa*, *-8ab*, *-8ba* and *-8bb*-type genes in teleost fishes [8,9,39]. Due to the absence of broad sampling of the most basal lineages of fishes, including hagfishes (Myxini), lampreys (Hyperoartia), cartilaginous fishes (Chondrichthyes), bichirs (Cladistia), sturgeons and paddlefishes (Chondrostei), as well as the most basal cohorts of teleosts (Elopomorpha and Osteoglossomorpha), it has remained unclear when the multigene *aqp8*-type system evolved in the actinopterygian lineage and whether other forms of *AQP8*-type genes might exist in vertebrates. Similarly, although the genomes of basal deuterostome lineages including sea urchins (Echinodermata), lancelets (Cephalochordata) and sea squirts (Tunicata) all encode one or more *aqp8*-type channel, it has not been established why chondrichthyan genomes appeared to lack such orthologs [39]. This latter observation is even more intriguing, given that a recent study identified an *aqp8* ortholog in the spiny dogfish shark (*Squalus acanthias*) [22].

To address these questions, here, we conducted Bayesian phylogenomic and syntenic analyses of *aqp8*-type CDS sampled from the major lineages of jawless (Agnatha) and jawed (Gnathostomata) vertebrates. Our data reveal that actinopterygian *aqp8aa, and -ab* genes evolved at chromosomal breakpoints with canonical orthologs of tetrapod *AQP8* now detected in all classes of vertebrate except hagfishes (Myxini). Although intact *aqp8* orthologs are detected in several squalomorph sharks, the majority of chondrichthyan genomes either lack *aqp8* CDS or retain fractionated pseudogenes. Conversely, we uncovered additional duplicates in Strepsirrhini primates as well as the canonical ortholog of tetrapod *AQP8* in basal lineages of actinopterygian fishes. This canonical *aqp8* ortholog underwent gene translocation in the common ancestor of Actinopterygii but was subsequently lost in the majority of teleost lineages. At or around the same time as the gene translocation event, co-orthologous tandemly arranged *aqp8aa*-*aqp8ab* duplicates were also generated in the original syntenic locus in the common ancestor of Actinopterygii. These latter co-orthologs maintained upstream synteny with the canonical *aqp8* loci of chondrichthyans and sarcopterygians and further expanded in teleosts via whole genome duplication (WGD) to form *aqp8ba-aqp8bb* and the tetraparalogous *aqp8* gene system. Taken together the present datasets provide a revised insight into the diversification of *aqp8*-type genes in vertebrates.

## 2. Results

### 2.1. Canonical AQP8 Orthologs Exist in All Vertebrate Lineages

**Figure 1:**
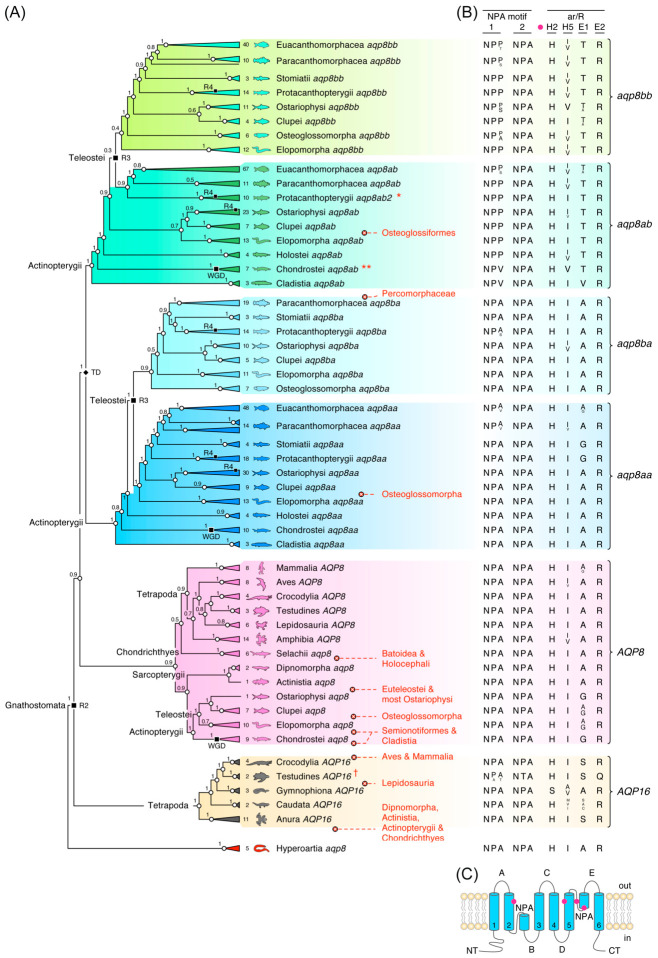
**Six major AQP8-related subtypes diverged in vertebrates.** (**A**) Summarized Bayesian majority rule consensus tree of canonical and non-canonical vertebrate *AQP8* orthologs rooted with hyperoartian *aqp8*. The tree is inferred from 60 million MCMC generations (nucmodel = 4by4, nst = 2, rates = gamma) of 564,230 nucleotide sites aligned by codon (N = 571 taxa). The number of taxa included in each collapsed subcluster is indicated. Tandem (TD) and whole genome duplication events (R3, R3, R4) are indicated at relevant nodes. Support values shown at each node are Bayesian posterior probabilities. Apparent absences of orthologs and co-orthologs are indicated for different taxonomic lineages in red. † indicates pseudogene. * R4-generated *aqp8ab1* pseudogenes not included. ** WGD-generated *aqp8ab2* pseudogenes not included. (**B**) Conservation or substitution of the NPA and ar/R residues scaled according to relative prevalence. (**C**) Schematic representation of an AQP8 integral membrane protein channel showing the six transmembrane domains, five loops, intracellular N- and C-termini and the location of the NPA motifs and ar/R residues (red dots). The fully annotated tree is shown in Supplementary Figure S1.

Initial phylogenetic analyses consisted of 572 *AQP8*-type CDS assembled from 220 genomes and 40 transcriptomes representing all of the major vertebrate classes and superclasses (Myxini, Hyperoartia, Chondrichthyes, Actinopterygii and Sarcopterygii). No *aqp8*-type CDS were detected in Myxini, but single-copy *aqp8* gene products were identified in lampreys (Hyperoartia) (Figure 1A; Supplementary Figure S1). These latter CDS were used to root the tree, which separated into six major subclusters consisting of *AQP16* in Tetrapoda, canonical *AQP8* in Gnathostomata and the tetraparalogous *aqp8aa-aqp8ab*, *aqp8ba-aqp8bb* system in Actinopterygii. As previously observed [39], *AQP16* CDS were only detected in Amphibia, Testudines and Crocodylia. In the present context, however, the data show that all three orders of Amphibia (Anura, Caudata and Gymnophiona) retain the *AQP16* genes and confirm that the orthologs are fractionated into pseudogenes in Testudines but appear functional in Crocodylia and Amphibia. Although no *aqp16* orthologs were identified in Actinopterygii, a surprising discovery was the first identification of canonical orthologs of tetrapod *AQP8* in this superclass of fishes. This included representatives of the Chondrostei, Elopomorpha, Clupei and a single copy in the milkfish (*Chanos chanos*), a basal member of the Ostariophysi. The tree further confirmed that the spiny dogfish shark sequence identified by Cutler and colleagues [22] is a canonical *aqp8* ortholog that co-clusters with five other chondrichthyan *aqp8* CDS. Two of these latter CDS appear to be respectively formed from intact genes in the Puget Sound dogfish shark (*Squalus suckleyi*) and the sharpnose sevengill shark (*Heptranchius perlo*), while the other sequences were derived from fractionated pseudogenes. The clustering of the chondrichthyan *aqp8* CDS is consistent with their higher identities (59 ± 4.1%; N = 3) with those of tetrapods (63% ± 0.5%; N = 44) when compared to the human AQP8 CDS. Conversely, actinopterygian *aqp8* CDS identities are lower (49 ± 2.3%; N = 28) in this respect.

The tree further revealed that the *aqp8aa-aqp8ab* binary gene cluster, previously only observed in the holostean spotted gar (*Lepisosteus oculatus*) and teleosts [39], is present in Cladistia, Chondrostei, and other holosteans, but it is not found in the genomes of Chondrichthyes or Sarcopterygii. The present data thus confirm that the *aqp8aa*-*aqp8ab* binary gene cluster was inherited by Teleostei but further indicate that it may have been lost in the majority of the Osteoglossomorpha. Conversely the *aqp8ba*-*aqp8bb* gene cluster is restricted to Teleostei, but with large-scale loss of the *aqp8ba* orthologs in the highly diverse percomorph teleosts. Taken together, this initial analysis suggested that canonical *AQP8* genes were inherited by the common ancestor of vertebrates but may have been lost in Myxini. Based upon the phylogenetic topology in Figure 1A and synteny analyses (Supplementary Figure S2), *AQP16* genes appear to have arisen at the R2 whole genome duplication (WGD) event in the common ancestor of Gnathostomata, which, together with the canonical *AQP8* genes, were either differentially retained or lost during evolution of the descendent lineages. Contrary to previous reports, however, the additional *aqp8aa* and *aqp8ab* genes are not direct orthologs of mammalian AQP8 but represent co-orthologs that selectively arose in the common ancestor of the Actinopterygii, and subsequently expanded to generate the teleost-specific *aqp8ba*-*aqp8bb* system following a tertiary round (R3) of WGD at the root of the crown clade.

These observations appear to be consistent with the diversification of the two NPA motifs and ar/R residues (Figure 1B,C). For intact genes, both NPA motifs are conserved between AQP16, canonical AQP8, Aqp8aa and Aqp8ba channels, with low rates of substitution of Ala to Val (NPV) at the tertiary position of the first NPA motif in Aqp8aa of some Paracanthomorphacea (cod-related teleosts) and Euacanthomorphacea (true spiny ray-finned teleosts) and Ala to Ser (NPS) in Aqp8ba of the Protacanthopterygii (salmon-related teleosts). Conversely, the tertiary position of the first NPA motif is mostly substituted to a Pro (NPP) or sometimes a Val (NPV) or Ser (NPS) in the Aqp8ab and Aqp8bb channels. Similar levels of diversification are also seen in the ar/R selectivity filter, which respectively show conserved His Ile/Val, Ala/Gly and Arg (H ^I^_V_^A^_G_ R) residues in the TMD H2, H5 and loop E1 and E2 positions of canonical AQP8, Aqp8aa and Aqp8ba channels and predominantly His, Ile/Val, Thr, Arg (H ^I^_V_ T R) residues in the Aqp8ab and Aqp8bb channels. Larger variance is seen in the intact AQP16 channels.

### 2.2. Pseudogenes Confirm the Loss of AQP8-Type Orthologs in Piscine Genomes

**Figure 2:**
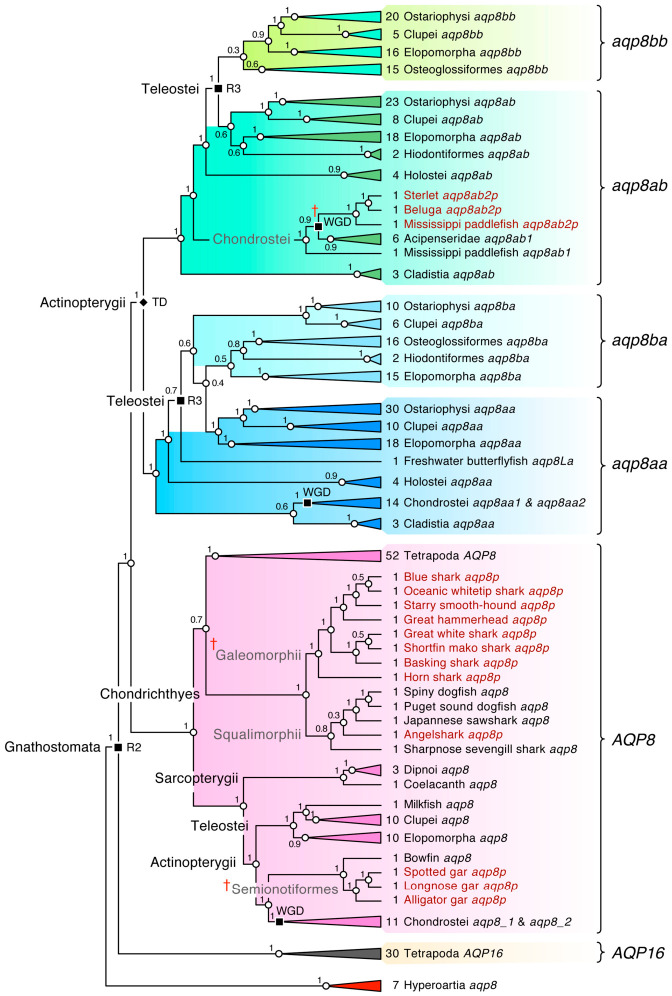
**Pseudogenes confirm lineage-specific inactivation/loss of gnathostome *aqp8*-type genes.** Summarized Bayesian majority rule consensus tree rooted with hyperoartian *aqp8*. The tree is inferred from 80 million MCMC generations (nucmodel = 4by4, nst = 2, rates = gamma) of 385,321 nucleotide sites aligned by codon (N = 383 taxa). The number of taxa included in each collapsed cluster is indicated. Tandem (TD) and whole genome duplication (WGD, R2, R3) events are indicated at relevant nodes. Support values shown at each node are Bayesian posterior probabilities. Pseudogenes of canonical *aqp8* and co-orthologous *aqp8ab2* are indicated in red text. † indicates grouped pseudogenes. The fully annotated tree including the Testudines *AQP16* pseudogenes is shown in Supplementary Figure S2.

**Figure 3:**
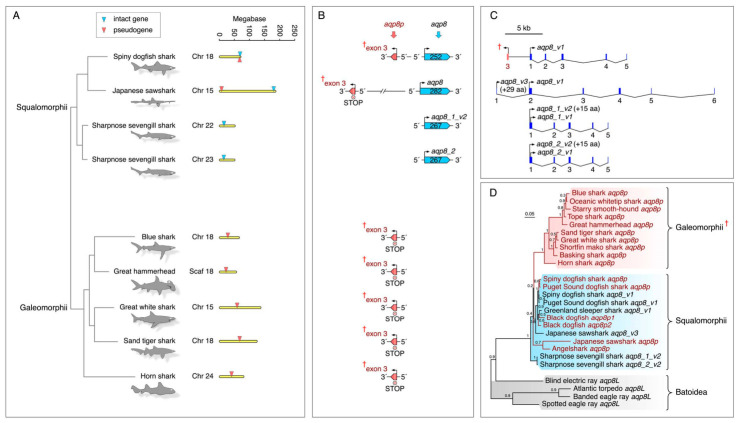
**Orthologous *aqp8* genes and pseudogenes of Chondrichthyes.** (**A**) Chromosomal loci of intact and fractionated *aqp8* genes in Selachii. (**B**) Genomic arrangement of *aqp8* genes and pseudogenes in Selachii. The pointed end of the gene symbols indicates the DNA coding strand with the number of amino acids of the intact translated proteins inside. (**C**) Gene structures of intact selachian *aqp8* orthologs, with alternative isoform start codons indicated. (**D**) Bayesian majority rule consensus tree of elasmobranch *aqp8* coding sequences. The tree is midpoint rooted and inferred from 500,000 thousand MCMC generations (nucmodel = 4by4, nst = 2, rates = gamma) of 22,354 nucleotide sites aligned by codon (N = 26 taxa). Support values shown at each node are Bayesian posterior probabilities. Pseudogenes are indicated in red text. † indicates grouped pseudogenes.

The absence of taxonomic representation of *AQP8*-type CDS is shown in Figure 1A. For canonical *aqp8* genes, this included the Batoidea (skates and rays) and Holocephali (ratfishes and ghost sharks), Euteleostei, most Ostariophysi, Osteoglossomorpha, Semionotiformes (gars) and Cladistia (bichirs). Similarly for the *aqp8aa-aqp8ab*, *aqp8ba-aqp8bb* gene systems, osteoglossomorph *aqp8aa*, *aqp8ab* and percomorph *aqp8ba* CDS were respectively lacking. Such absences could be the result of incomplete genome sequencing rather than gene loss, and we therefore resampled the genomes to identify possible pseudogenes of the above taxonomic groups to verify the earlier observations. In this respect, we resampled the genomes of 69 Chondrichthyes (27 Selachii, 37 Batoidea and 5 Holocephali), four Holostei and 33 Osteoglossomorpha (2 Hiodontiformes and 31 Osteoglossiformes). Bayesian inference of the assembled sequences was performed on all taxa except euteleostean sequences in an effort to maximize nodal posterior probabilities (PP). The results revealed that intact chondrichthyan *aqp8* orthologs are currently only found in the Selachii and more specifically the squalomorph sharks, while the galeomorph sharks only retain pseudogene remnants (Figure 2; Supplementary Figure S3). No *aqp8*-related sequences were identified in the genomes of the Holocephali; however, several isolated *aqp8*-like exons were detected in four species of batoid rays (Figure 3). Analysis of the selachian *aqp8* genes showed that, even in species that retain intact orthologs, such as the spiny dogfish shark and the Japanese sawshark (*Pristiophorus japonicus*), upstream pseudogenes also exist on the opposing DNA strand (Figure 3A,B). In the case of the spiny dogfish shark, the counter-coding pseudogene remnant is located immediately upstream in the 5′ region of the intact *aqp8* gene, which, as for many gnathostome orthologs, is composed of five exons (Figure 3C). In the Japanese sawfish, however, the pseudogene is located at the opposing end of the chromosome. All pseudogenes detected in the Selachii correspond to an exon 3 region as defined by the spiny dogfish shark and sharpnose sevengill shark (*Heptranchius perlo*) *aqp8* gene structures. Despite the relatively short length and degraded nature of the pseudogenes, Bayesian inference shows that they cluster within the separate galeomorph and squalomorph subclades, while the batoid *aqp8*-like exons cluster separately (Figure 3D; Supplementary Figure S4). An additional feature noted for the intact squalomorph Aqp8 channels was the existence of alternative N-terminal splice variants. The shortest isoforms (Aqp8_v1) are encoded by five exons as represented by the spiny and Puget Sound dogfish sharks with 252 amino acids (aa), while the duplicates of the sharpnose sevengill shark (Aqp8_1_v2 and Aqp8_2_v2), also encoded by five exons, have an additional in-frame start codon that extends the N-terminus by 15 aa. Conversely, the Aqp8_v3 channel of the Japanese sawshark is extended by 30 aa due to the splicing of an additional upstream exon 1. As a result, the Japanese sawshark Aqp8_v3 variant is encoded by six exons. Such additional exons also generate N-terminal splice variants in mammalian and piscine AQP8 channels.

For basal actinopterygian fishes, we identified an intact canonical *aqp8* CDS in the bowfin (*Amia calva*), but only fractionated pseudogenes in the three semionotiform gar genomes (Figure 2). In the Chondrostei, additional *aqp8ab2* pseudogenes were also identified as duplicates of the *aqp8ab1* genes, which together with duplicated *aqp8_1*, *aqp8_2*, *aqp8aa1* and *aqp8aa2* genes, all of which are located on separate chromosomes, are consistent with an independent polyploidy event in the lineage [40,41]. No further canonical *aqp8* sequences were detected in Cladistia, Osteoglossomorpha, most Ostariophysi or Euteleostei, suggesting that they may be lost in these lineages.

### 2.3. Differential Retention of the aqp8 Binary Gene Clusters in Osteoglossomorpha

**Figure 4:**
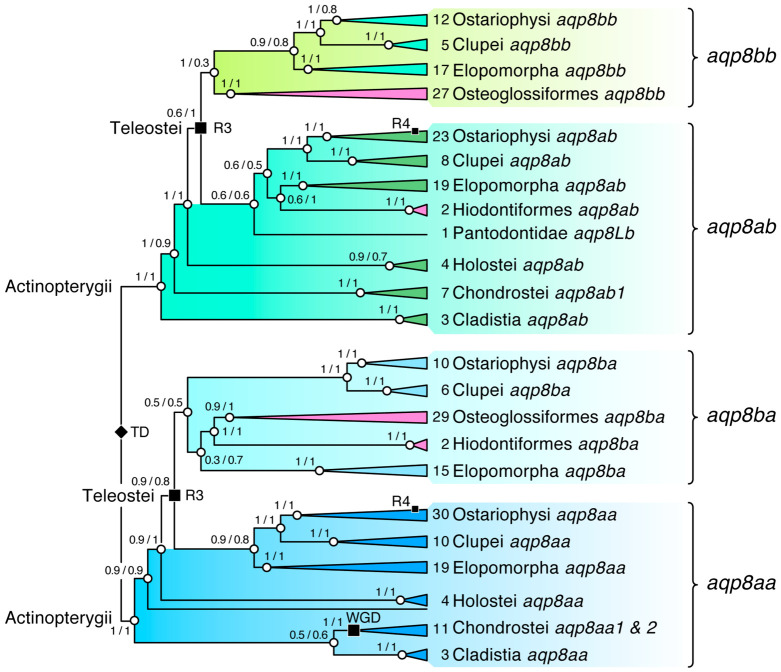
**Differential retention of actinopterygian *aqp8*-type binary cluster genes in elopomorph, osteoglossomorph and otomorph teleosts.** Combined Bayesian majority rule consensus tree with and without the N-termini inferred from 15 million MCMC generations (nucmodel = 4by4, nst = 2, rates = gamma). The tree is midpoint rooted and calculated from of 233,336 nucleotide sites (full length) and 185,635 nucleotide sites (N-terminally truncated) aligned by codon (N = 268 taxa). The number of taxa included in each collapsed cluster is indicated. Tandem (TD) and whole genome duplication events (WGD, R3, R4) are indicated at relevant nodes. Support values shown at each node are Bayesian posterior probabilities for full length/N-terminally truncated trees. The fully annotated tree is shown in Supplementary Figure S4.

The Osteoglossomorpha are comprised of two orders, the Hiodontiformes with a single family of goldeyes and mooneyes and the more diverse Osteoglossiformes with five families of bonytongues. In contrast to the sister clade of Elopomorpha, which retain five *aqp8*-type genes, including canonical *aqp8* and the two *aqp8aa*-*aqp8ab* and *aqp8ba*-*aqp8bb* binary gene clusters, the initial analyses indicated that the Osteoglossomorpha may only retain the *aqp8ba*-*aqp8bb* gene cluster (Figure 1A). However, the second analysis indicated that the hiodontiform genomes have differentially retained the *aqp8ab* and *aqp8ba* genes, while the genomes of the bonytongues only retain the *aqp8ba*-*aqp8bb* binary gene cluster (Figure 2). To verify these observations, we extended the sampling of osteoglossomorph genomes to include all six families with each of the 21 genera of the highly diverse Mormyridae represented. The assembled *aqp8aa*, *aqp8ab*, *aqp8ba* and *aqp8bb* CDS were analyzed via Bayesian inference in relation to those of the Cladistia, Chondrostei, Holostei, Elopomorpha and Otomorpha, both as complete codon alignments without any pseudogene products and also following removal of the N-termini from the alignments. The resultant majority rule consensus trees were midpoint rooted in order to avoid potential biases that could be introduced by the long branch of the hyperoartian root. The combined results provided good statistical support for the previous analyses with separation of the actinopterygian *aqp8aa* and *aqp8ab* paralogs in the preteleost lineages and the tetraparalogous teleost *aqp8aa*, *aqp8ab*, *aqp8ba* and *aqp8bb* CDS supported by relatively high PP (Figure 4; Supplementary Figure S5). The data thus suggest that the hiodontiform genomes indeed no longer possess gene clusters with only *aqp8ab* and *aqp8ba* genes retained, while those of the Osteoglossiformes mostly encode the *aqp8ba*-*aqp8bb* binary gene cluster. An exception could be the freshwater butterflyfish (*Pantodon buchholzi*, Pantodontidae, Osteoglossiformes), two transcripts of which showed differential clustering with varying PP in each of the trees. These transcripts were therefore annotated as *aqp8La* and *aqp8Lb*, respectively.

### 2.4. Synteny Reveals Gene Translocation of Canonical aqp8 in Actinopterygii

**Figure 5:**
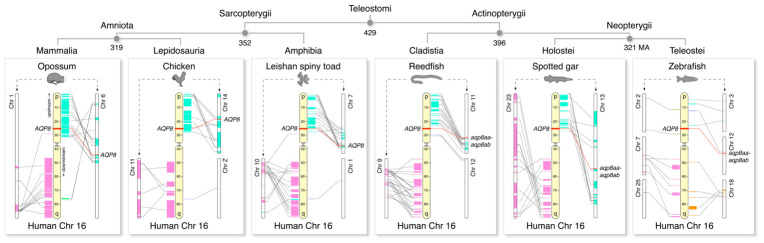
**Macrosynteny of gnathostome *AQP8*-type loci reveals human chromosome 16 arose via fusion.** Major regions of two Euteleostomi chromosomes highlighted in light green and pink respectively map to the p and q arms of human chromosome 16. *AQP8*-type gene loci are highlighted in red with subregion mapping indicated by black lines. Divergence times represent median values from time tree (Kumar et al., 2022) [42].

**Figure 6:**
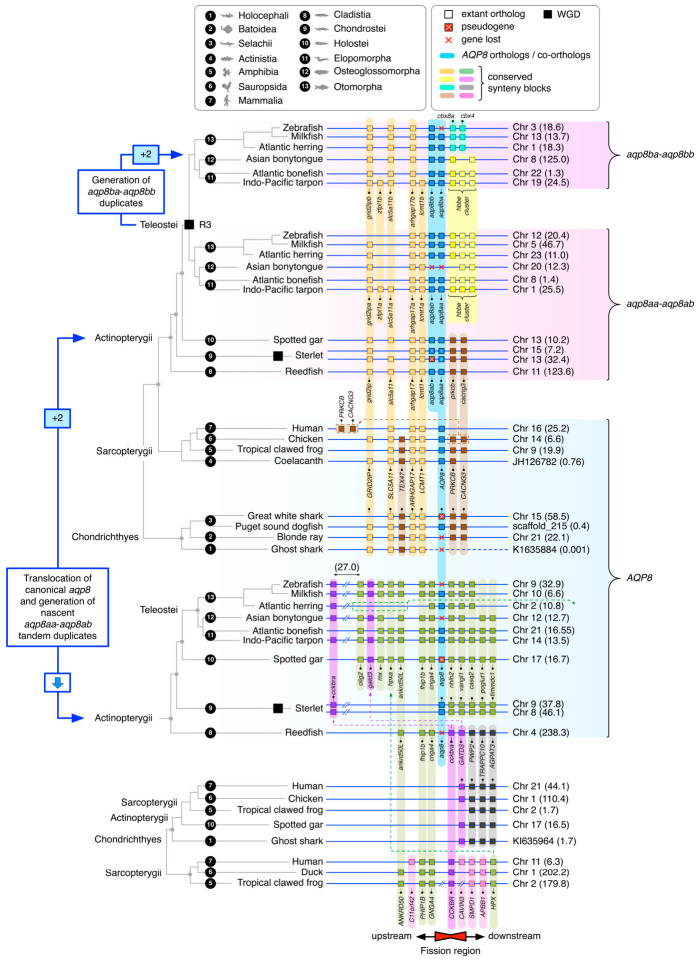
**Microsynteny reveals *aqp8* gene translocation and reciprocal *aqp8*-type binary gene cluster generation in Actinopterygii.** Syntenic arrangement of *AQP8*-type genes in Gnathostomata illustrating conservation of flanking genes (colored blocks) between chondrichthyan and sarcopterygian canonical *aqp8* loci and actinopterygian *aqp8*-type binary gene clusters as well as translocated canonical *aqp8* loci in actinopterygians. Upstream and downstream segments are defined according to the locus of human *AQP8*.

A macrosynteny analysis in relation to the location of human *AQP8* at 25.2 Mb on Chromosome 16 shows that, despite lineage-specific rearrangements, major regions of the short (p) and long (q) arms of chromosome 16 can be mapped to two separate chromosomes over >400 million years of evolution (Figure 5). These data thus suggest that human chromosome 16 arose via fusion of two chromosomes and that the *AQP8* gene locus is conserved within the orthologous sarcopterygian regions that map to the p arm. Conversely, although major portions of two actinopterygian chromosomes also consistently map to the p and q arms of human chromosome 16, the canonical *aqp8* gene locus is replaced by the binary *aqp8aa-aqp8ab* gene cluster in the syntenic actinopterygian chromosome. This latter gene replacement is also observable in teleost genomes, as represented by the zebrafish; however, only very low levels of macrosynteny remain. A detailed microsynteny analysis confirmed these observations and revealed that the upstream and downstream flanking genes of canonical *AQP8* show conserved synteny between the genomes of Sarcopterygii and Chondrichthyes, with only conservation of the upstream flanking genes in Actinopterygii (Figure 6). In all actinopterygian genomes, the canonical *AQP8* genes are translocated to a new genomic environment that is not syntenic with the genomes of the Sarcopterygii or Chondrichthyes, while the binary *aqp8aa-aqp8ab* and *aqp8ba-aqp8bb* gene clusters exist in the original syntenic locus.

Amongst the most basal actinopterygians is the cladistian reedfish (*Erpetoichthys calabaricus*), which has conserved upstream flanking gene synteny to the canonical *aqp8* gene locus of other actinopterygians and a conserved downstream flanking gene synteny to Sarcopterygii and Chondrichthyes. Three central genes, including canonical *aqp8*, cholecystokinin B receptor (*cckbr*) and glutamine amidotransferase class 1 domain-containing 3 (*gatd3*) are the anchors of each of the above conserved synteny regions. This suggests that this region of the chromosome, exemplified by reedfish chromosome 4 locus 238.3 Mb, represents a fission region and that canonical *aqp8* underwent gene translocation in the ancestor of Actinopterygii. The data further suggest that either coincident with this translocation event or soon after, the actinopterygian *aqp8aa-aqp8ab* binary gene cluster was established in the original locus.

### 2.5. AQP8 Is Duplicated in the Strepsirrhini Primates

**Figure 7:**
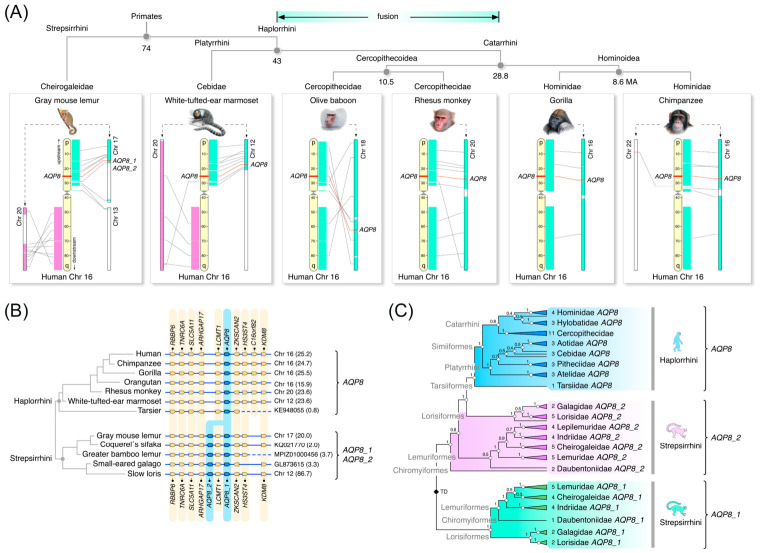
***AQP8* is duplicated in Strepsirrhini primates.** (**A**) Macrosynteny of primate *AQP8* loci indicating that the fusion event that formed human chromosome 16 occurred during a ~14 million window after separation of the Catarrhini from the Platyrrhini. Divergence times represent median values from time tree [42]. (**B**) Microsynteny of *AQP8* and duplicated *AQP8_2* loci in primates. The 5′-3′coding direction is illustrated by the pointed end of the gene symbol. (**C**) Summarized Bayesian majority rule consensus tree of primate *AQP8*-type CDS inferred from 1 million MCMC generations (nucmodel = 4by4, nst = 2, rates = gamma). The tree is midpoint rooted and inferred from of 57,949 nucleotide sites aligned by codon (N = 76 taxa). Support values shown at each node are Bayesian posterior probabilities. TD: tandem duplication. The fully annotated tree is shown in Supplementary Figure S5. Primate images are reproduced with the permission of Jón Baldur Hlidberg.

During the course of the synteny analyses described above, we noted that some primates appeared to have additional *AQP8* paralogs. To understand whether this was the result of a common ancestral duplication event or represented independent lineage-specific duplications, we investigated these possibilities in the primate lineage. Macrosynteny analyses of the gene loci in relation to that of human *AQP8* revealed that major portions of chromosomes 17 and 20 of the gray mouse lemur (*Microcebus murinus*; Strepsirrhini) and chromosomes 12 and 20 of the white-tufted-ear marmoset (*Callithrix jacchus*, Platyrrhini) respectively map to the p and q arms of human chromosome 16. Conversely, amongst the linkage groups of the Catarrhini, only single chromosomes map to the full length of human chromosome 16 (Figure 7A). These observations suggest that the chromosomal fusion event that formed the precursor of human chromosome 16 apparently occurred during a ~14-million-year window in the common ancestor of the Catarrhini after the lineage separated from the Platyrrhini.

The microsynteny analyses show that a second *AQP8* paralog (*AQP8_2*) exists in the genomes of Strepsirrhini, which is located between the upstream leucine carboxyl methyltransferase 1 (*LCMT1*) and rho GTPase-activating protein 17 (*ARHGAP17*) genes on the opposing DNA strand (Figure 7B). To confirm whether the *AQP8_2* genes are restricted to the Strepsirrhini, we assembled 76 *AQP8* CDS from 15 of the 16 families of primates, since genomic data from the last family of sloth lemurs (Palaeopropithecidae) are currently not available. Amongst the 76 assembled CDS, we noted that the aye-aye (*Daubentonia madagascariensis*, family: Daubentoniidae) encodes a ternary cluster of three *AQP8* paralogs, while the Moholi bushbaby (*Galago moholi*, family: Galagidae) also encodes three *AQP8* paralogs with the third representing a pseudogene. Additional *AQP8* pseudogenes were also identified in the genomes of the slender loris (*Loris tardigradus*) and the Bengal slow loris (*Nycticebus bengalensis*), both members of the Lorisidae family. Bayesian inference of the aligned codons, excluding the Moholi bushbaby pseudogene, showed that *AQP8_2* gene duplicates are indeed restricted to the Strepsirrhini (Figure 7C; Supplementary Figure S6). The tree topology suggests, however, that the Strepsirrhini *AQP8_2* CDS are more closely related to the Haplorrhini *AQP8* orthologs, consistent with a relatively lower nucleotide substitution rate in relation to human *AQP8* of the *AQP8_2* CDS (12.5 ± 0.7%) compared to the AQP8_1 CDS (19.2 ± 1.2%).

## 3. Discussion

In the present work, we show that the canonical orthologs of the mammalian *AQP8* exist in agnathan lampreys and the three major divisions of Gnathostomata, namely the Chondrichthyes, Actinopterygii and Sarcopterygii. This finding was unexpected, since previous studies had considered the *aqp8aa*, *aqp8ab*, *aqp8ba* and *aqp8bb* genes to be the only orthologs encoded in actinopterygian genomes (reviewed by [43]. The current phylogenomic data nevertheless show that, in addition to these *aqp8aa-aqp8ab* and *aqp8ba-aqp8bb* binary gene clusters, the canonical *aqp8* orthologs also exist in preteleost chondrosteans and holosteans as well as teleost elopomorphs, clupeids and a basal ostariophysan, the milkfish. Available transcriptomic data further confirm that these latter canonical *aqp8* orthologs are expressed in the chondrosteans, elopomorphs and clupeids and are thus functional at the transcriptional level. The actinopterygian canonical *aqp8* gene loci are not, however, syntenic with the orthologous *aqp8* loci in sarcopterygians or chondrichthyans, but are found in an entirely new genomic environment, while the *aqp8aa-aqp8ab* binary gene clusters of all actinopterygians are syntenic with the upstream flanking genes of the *aqp8* genes in sarcopterygians and chondrichthyans. This not only suggests that the canonical *aqp8* gene was translocated in the common ancestor of the Actinopterygii, but that the *aqp8aa-aqp8ab* binary cluster was generated in close temporal proximity to this event. Insight as to how this might have occurred arises from studies of chromosomal evolution in the fungal human pathogen *Cryptococcus neoformans* [44]. In a congenic strain of this organism, it was shown that two chromosomes underwent fusion, fission and translocation events to generate novel genetic linkage maps sharing segmental duplications. The authors hypothesized that such chromosomal recombinations may have occurred during meiosis and highlighted similar processes in yeast, cancer cells and the formation of human chromosome 2 [44]. An important consequence is the generation of novel genes that may confer evolutionary potential, where at least one of the genes can maintain the original function, while others are less constrained and can evolve novel functions [45]. In the case of the common ancestor of the Actinopterygii, the putative recombinatory events could have led to the existence of four *aqp8*-type genes, canonical *aqp8* together with *aqp8aa*, *aqp8ab* and *aqp16*. There is currently no evidence for *aqp16* in actinopterygian genomes; however, the syntenic analyses (Supplementary Figure S2) suggest that *AQP16* indeed may have arisen during the R2 WGD event but was lost during early actinopterygian evolution. This left three extant *aqp8-type* genes (canonical *aqp8* and the *aqp8aa*-*aqp8ab* cluster) in the common ancestor of Actinopterygii that were inherited by Teleostei. The subsequent teleost-specific R3 WGD event should therefore have generated six *aqp8*-type genes, but as evidenced by the molecular phylogenetic and syntenic data, only the R3-generated *aqp8ba-aqp8bb* gene cluster survived, while the duplicated canonical *aqp8* did not. This scenario leaves the five teleost *aqp8*-type genes (canonical *aqp8*, and the two *aqp8aa-aqp8ab* and *aqp8ba-aqp8bb* gene clusters) shown here in the Elopomorpha, Clupei and the ostariophysan milkfish.

The molecular basis for the preferential retention of the *aqp8*-type binary gene clusters rather than the canonical *aqp8* orthologs in the majority of actinopterygians is not known; however, it can be speculated that gene translocation to a new genomic environment may not include enhancers and other cis- or trans-regulatory elements [46]. By remaining in the original genomic environment, the control elements would contribute to the regulation of the new occupants, i.e., the binary *aqp8*-type gene clusters, such that functional continuity of at least one of the genes is maintained, while the translocated progenitor is rendered redundant. Conversely, the new nuclear organization of the translocated gene can result in altered expression dynamics, a feature associated with oncogenesis and cancer [47,48]. Such mechanisms do not, however, seem compatible with the loss of *aqp8* orthologs in many chondrichthyans, since the microsynteny data show no evidence of gene translocation. Thus, although our data for *aqp8* genes in Chondrichthyes confirm the recent finding of the channel in the spiny dogfish shark [22], they also reveal that intact canonical *aqp8* orthologs are currently only found in selachian sharks, with functional CDS so far restricted to the Squalomorphii. The present discovery of pseudogenes in both squalomorph and galeomorph sharks also explains why *aqp8* orthologs previously remained undetected and suggests that there seems to have been widespread inactivation or loss of *aqp8* genes in many lineages of Chondrichthyes. Contrary to this notion, however, is the present identification of several *aqp8*-like exons in isolated contigs of some batoid rays, which could indicate that the *aqp8* genes may reside in dark regions of the genome that are currently difficult to sequence [49], and consequently, more complete sequencing could reveal a higher prevalence of these channels in the future. Moreover, since vertebrate Aqp8 channels are established as urea transporters [8,9,50,51], it seems surprising that selection pressure did not favor their retention in animals that maintain high concentrations of urea to counteract the osmotic pressure of seawater [52]. It has been suggested that Aqp8 does not play a homeostatic role in the gill, kidney or gastrointestinal tract of the spiny dogfish shark [22], but this is not known for other species of cartilaginous fishes. Chondrichthyes do, however, retain multiple transmembrane channels permeable to urea and ammonia including aquaglyceroporins (Aqp3, -9 and -10 subfamilies) and urea transporters (Slc14a subfamily) [53,54] that may have compensated for the seemingly widespread loss of *aqp8*.

The evolution of a novel *AQP8_2* gene in Strepsirrhini primates provides an example of the stochastic nature of gene evolution. The *AQP8_2* genomic locus is not directly syntenic with the *AQP8* orthologs of the Haplorrhini but shifted upstream between the *LCMT1* and *AHRGAP17* flanking genes. Nevertheless, the molecular phylogeny shows that the Strepsirrhini *AQP8_2* gene is more closely related to the Haplorrhini *AQP8* orthologs than the Strepsirrhini *AQP8_1* gene, which is directly syntenic to the Haplorrhini *AQP8* orthologs. The molecular phylogeny is consistent with the relatively lower rates of nucleotide substitution in the Strepsirrhini *AQP8_2* CDS and thus shows, in contrast to findings in *Drosophila* [55], that the post-duplication rate of neofunctionalization may affect the older progenitor rather than the younger duplicated gene.

## 4. Materials and Methods

**Sequence Assembly and Phylogenetic Analyses.** AQP8-type peptide sequences were obtained from open-source whole genome shotgun (WGS), transcriptome shotgun (TSA), nucleotide or protein databases via tblastn or blastp (https://blast.ncbi.nlm.nih.gov/Blast.cgi, https://www.genomeark.org/ accessed on 21 April 2026), Genome Warehouse (https://ngdc.cncb.ac.cn/gwh/, accessed on 21 April 2026) and https://asia.ensembl.org/index.html, accessed on 21 April 2026), as described previously [37,56]. For CDS assembly, either full-length proteins or exon-deduced peptides were used as queries. Full-length and partial proteins were then aligned to generate multiple sequence alignments using ClustalW v2.1 [57]. Corresponding nucleotide sequences were retrieved from the respective DNA contigs, linkage groups or databases and trimmed to match each peptide prior to conversion to codon alignments using Pal2Nal v14.1 [58]. Phylogenetic analyses were conducted via Mr Bayes v3.2.7a, with model parameters nucmodel = 4by4, nst = 2, rates = gamma [59], on the full-length codon alignments following removal of gapped regions containing a single sequence or following removal of the N-termini. Between 0.5 and 80 million Markov chain Monte Carlo (MCMC) generations were run with three heated and one cold chain, with the resulting posterior distributions examined for convergence, an effective sample size >1000 using Tracer version 1.7.1 [60] and majority rule consensus trees summarized with a burn in of 25%. The alignments and annotated trees are provided in the supplementary material.

Macro- and micro-synteny analyses were performed using Ensembl v115, Ensembl beta, and Genomicus [61] or manually via tblastn for the flanking genes. Gene structures were mapped against the corresponding DNA sequences using the exons. Pseudogenes identified with indels, premature stop codons and deletions were obtained manually by aligning DNA regions of the expected *AQP8*-type loci against related exon-deduced peptides or full length AQP8 orthologs. The taxonomic nomenclature follows the definitions at NCBI (www.ncbi.nlm.nih.gov/taxonomy, accessed on 21 April 2026). Interpretations of the phylogenetic interrelationships of vertebrates are based on Steiper and Young [62], Naylor et al. [63], and Hughes et al. [64].

## 5. Conclusions

The present findings provide new insight into the diversification of the *AQP8*-related gene family in vertebrates. The data reveal that canonical orthologs of tetrapod *AQP8*-type water channels likely existed in all classes of vertebrate but were possibly lost in Myxini (hagfishes). In chondrichthyan (cartilaginous) fishes, intact *aqp8* genes are currently only found in squalomorph sharks, but as evidenced through the identification of pseudogenes, they seem to have been inactivated and/or lost in the other lineages. In actinopterygian ray-finned fishes, the canonical ortholog of tetrapod *AQP8* appears to have undergone gene translocation in their common ancestor ~400 MYA and is currently only identified in preteleost chondrosteans and holosteans as well as basal groups of teleosts (elopomorphs, clupeids and gonorynchiforms). Conversely, the ray-finned fish *aqp8aa-aqp8ab* gene cluster evolved in the original syntenic locus possibly as a result of meiotic recombination. Synteny analyses suggest that the *AQP16* genes may have arisen as a consequence of the second round (R2) of whole genome duplication in vertebrates, while the *aqp8ba-aqp8bb* binary gene cluster resulted from the teleost-specific genome duplication (R3). Integration of the datasets thus suggest that chromosomal translocation, recombination and replication were important events underlying the diversification of vertebrate *AQP8*-type genes. In addition, the identification and analysis of duplicated *AQP8_2* genes in Strepsirrhini primates provides evidence for the notion that neofunctionalization can affect either the precursor or descendent gene. Such neofunctionalization is also noted in the NPA-motifs and ar/R selectivity filters of the teleost *aqp8*-type binary gene clusters. However, whether this contributed to the differential basis of glycerol and urea permeation of the different teleost Aqp8-type channels remains to be established.

## Data Availability

All relevant data can be found within the article and its supplementary information.

## Supplementary Materials

The following supporting information can be downloaded at https://www.mdpi.com/article/10.3390/ijms27093937/s1.
