# Supplementary material for: Chromosomal Replication, Translocation and Recombination as Putative Events in the Diversification of Vertebrate AQP8-Type Genes
Source: Int J Mol Sci. 2026 Apr 28;27(9):3937. doi: 10.3390/ijms27093937 (PMC13163493; doi:10.3390/ijms27093937)
Supplement: Supplementary file 1 [file ijms-27-03937-s001.zip › ijms-4232752-supplementary captions.pdf]

Supplementary Material

Figure S1. Annotated Bayesian majority rule consensus tree of vertebrate *AQP8*-related channels (Fig. 1 main text). The tree is rooted with hyperoartian lamprey *aqp8s* and inferred from 60 million MCMC generations (nucmodel = 4by4, nst = 2, rates = gamma) of 2564,230 nucleotide sites aligned by codon (N = 571 taxa). Support values shown at each node are Bayesian posterior probabilities. The scale bar indicates the expected rate of substitutions rate per site. Pseudogenes and apparent absences of orthologs and co-orthologs are indicated for different taxonomic lineages in red. Whole genome duplications (WGD, R2, R3, R4) and tandem duplications (TD) are indicated at relevant nodes.

Figure S2. Microsynteny of *AQP16* and *AQP8*-type genes. (A) Phyloview of *AQP16* microsynteny, with 5´-3´gene coding direction indicated by the pointed end of the gene symbol. Othologous genes are repesented by the same gene symbol colour, with the chromsomal loci (megabases) of the ARHGAP44 gene search query indicated in parentheses. Tropical clawed frog ARHGAP44 (ENSXETG00000019547) was used as the reference gene query. Grey, pink and green backgrounds highlight the conserved synteny blocks. R3 indicates the teleost-specific whole genome duplication. (B) Alignview of the syntenic relationships between *AQP16*, *AQP8*, *aqp8aa-aqp8ab* and *aqp8ba-aqp8bb*. R2 indicates the second round of vertebrate whole genome duplication. Gene query, symbol orientations, colours and backgrounds as in (A). A thick blue line between two genes is equivalent to a "gap" in the alignment of specific species where the two genes are neighbours in this species but not in the reference species. A thin line between two genes is equivalent to a "break" in the continuity of the alignment where the two genes are linked (on the same chromosome or scaffold) in the order shown in the corresponding species but at least one gene separates the two genes in that species. A thin double-headed arrow under a gene or block of genes means that the order of the genes shown was reversed compared to the "canonical" orientation found in Ensembl.

Figure S3. Annotated Bayesian majority rule consensus tree of vertebrate *AQP8*-related CDS and pseudogenes (Fig. 2 main text). The tree is rooted with hyperoartian lamprey *aqp8s* and inferred from 80 million MCMC generations (nucmodel = 4by4, nst = 2, rates = gamma) of 385,321 nucleotide sites aligned by codon (N = 383 taxa). Support values

shown at each node are Bayesian posterior probabilities. The scale bar indicates the expected rate of substitutions rate per site. Pseudogenes are indicated in red text. Whole genome duplications (WGD, R2, R3, R4) and tandem duplications (TD) are indicated at relevant nodes.

Figure S4. Annotated Bayesian majority rule consensus tree of chondrichthyan *aqp8* CDS and pseudogenes. (A) Fig. 3D main text. The tree is midpoint rooted and inferred from 500 thousand MCMC generations (nucmodel = 4by4, nst = 2, rates = gamma) of 14,612 nucleotide sites aligned by codon (N = 17 taxa). Support values shown at each node are Bayesian posterior probabilities. The scale bar indicates the expected rate of substitutions rate per site. Pseudogenes are indicated in red text.

Figure S5. Annotated Bayesian majority rule consensus tree of actinopterygian *aqp8*-type binary gene clusters (Fig. 4 main text). The tree is midpoint rooted and calculated from of 233,336 nucleotide sites (full length) and 185,635 nucleotide sites (N-terminally truncated) aligned by codon (N = 268 taxa). Support values shown at each node are Bayesian posterior probabilities for full length/N-terminally truncated alignments. The scale bar indicates the expected rate of substitutions rate per site. Whole genome duplications (WGD, R3, R4) and tandem duplications (TD) are indicated at relevant nodes.

Figure S6. Annotated Bayesian majority rule consensus tree of primate *AQP8*-type CDS (Fig. 7C main text). The tree is midpoint rooted and calculated from of 57,949 nucleotide sites aligned by codon (N = 76 taxa). Support values shown at each node are Bayesian posterior probabilities. The scale bar indicates the expected rate of substitutions rate per site. TD: tandem duplication.

Supplementary Data Files

File S1: Alignment for Fig. 1A (& Fig. S1)

File S2. Alignment for Fig. 2 (& Fig S3)

File S3. Alignment for Fig. 3C (& Fig S4A)

File S4. Alignment for Fig S4B

File S5. Alignment for Fig. 4 (& Fig S5)

File S6. Alignment for Fig. 7C (& Fig S6)
