# Supplementary figures and images for: Chromosomal Replication, Translocation and Recombination as Putative Events in the Diversification of Vertebrate AQP8-Type Genes
Source: Int J Mol Sci. 2026 Apr 28;27(9):3937. doi: 10.3390/ijms27093937 (PMC13163493; doi:10.3390/ijms27093937)

Figure S1

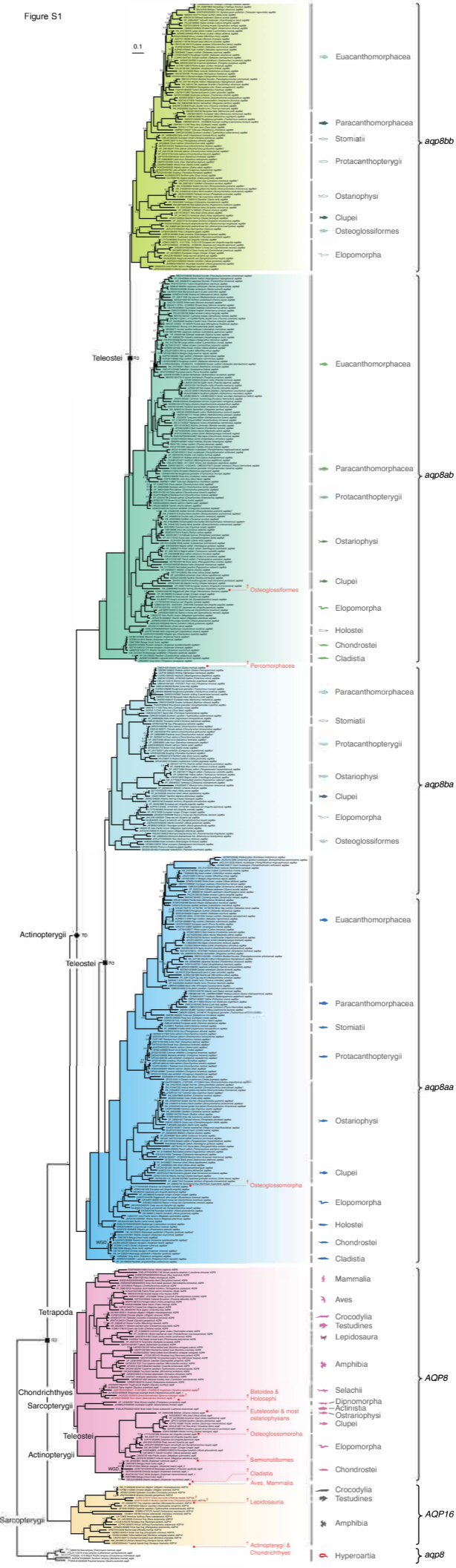

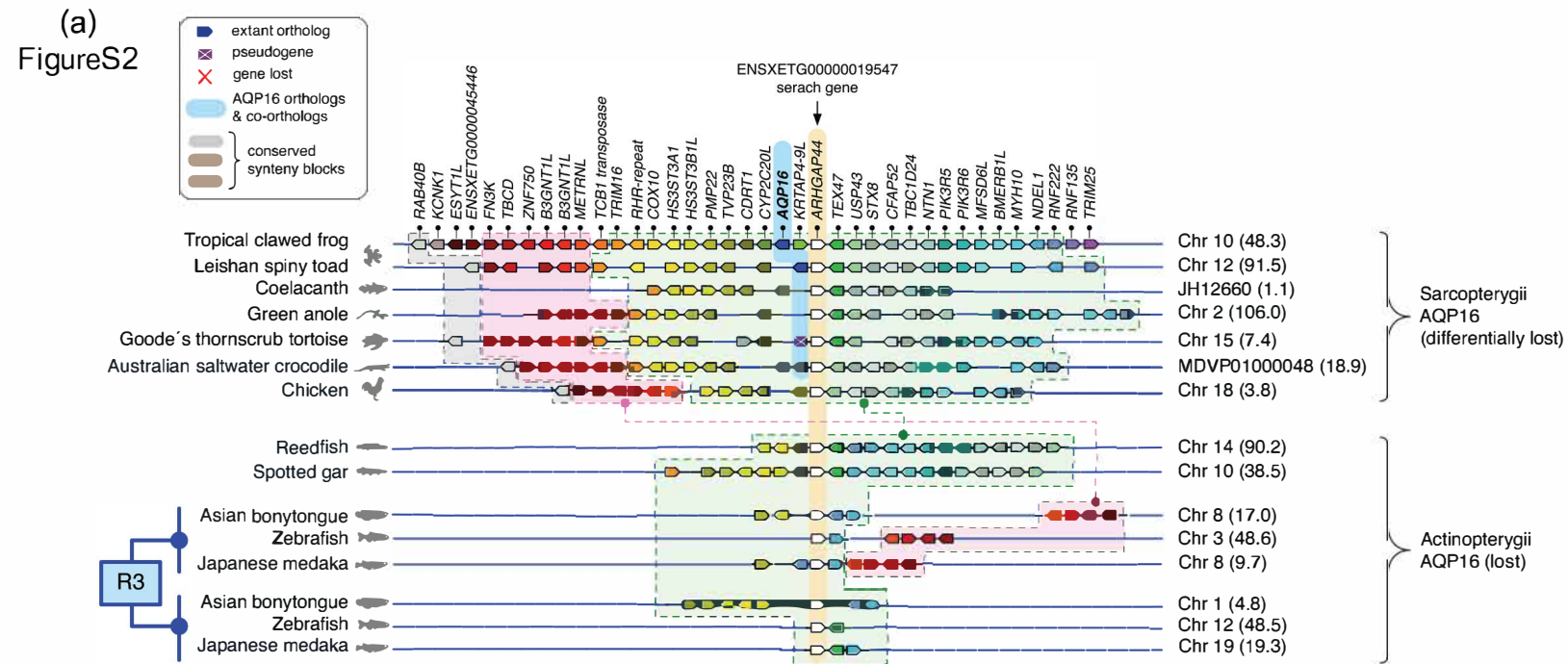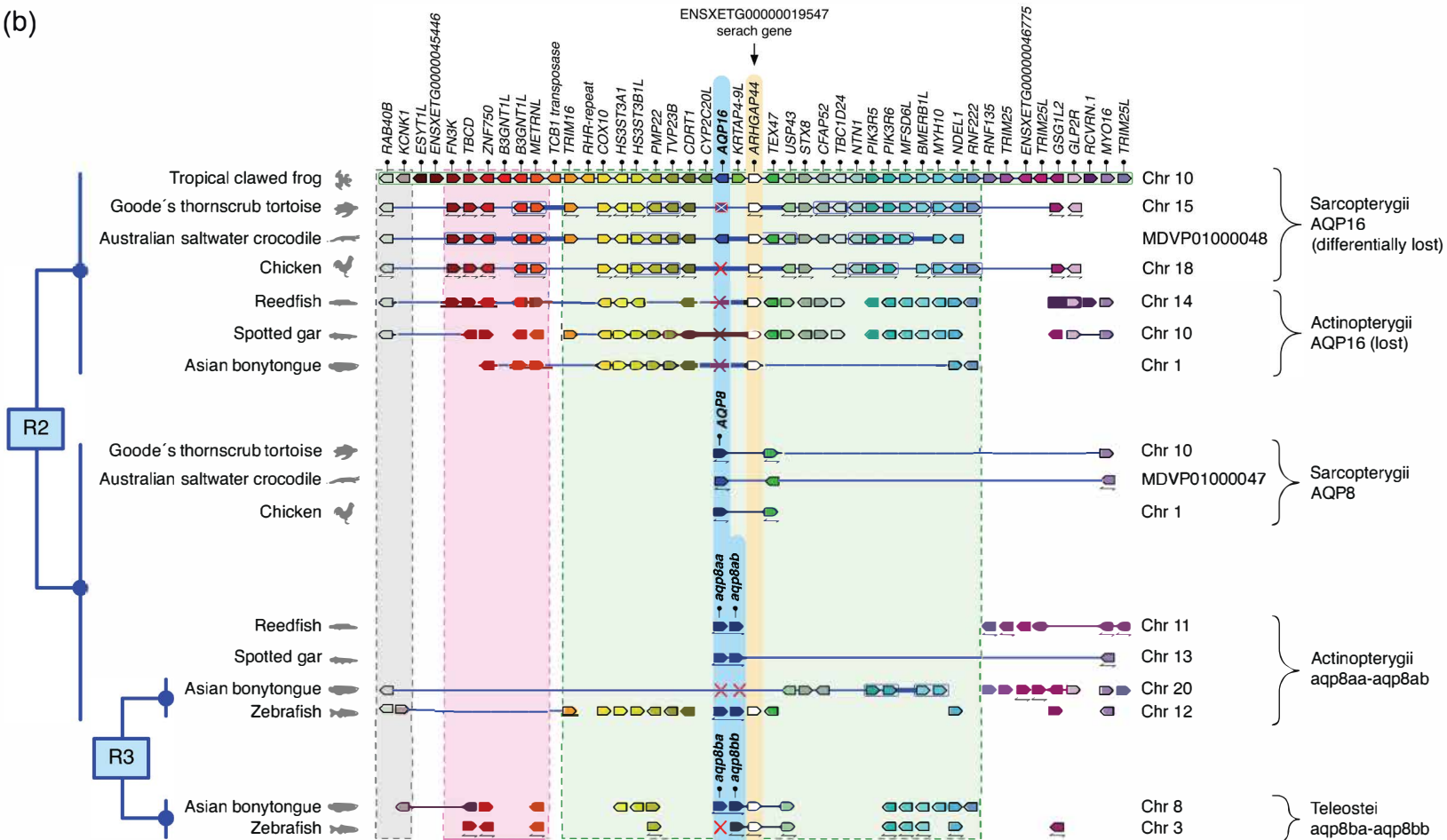

Figure S3

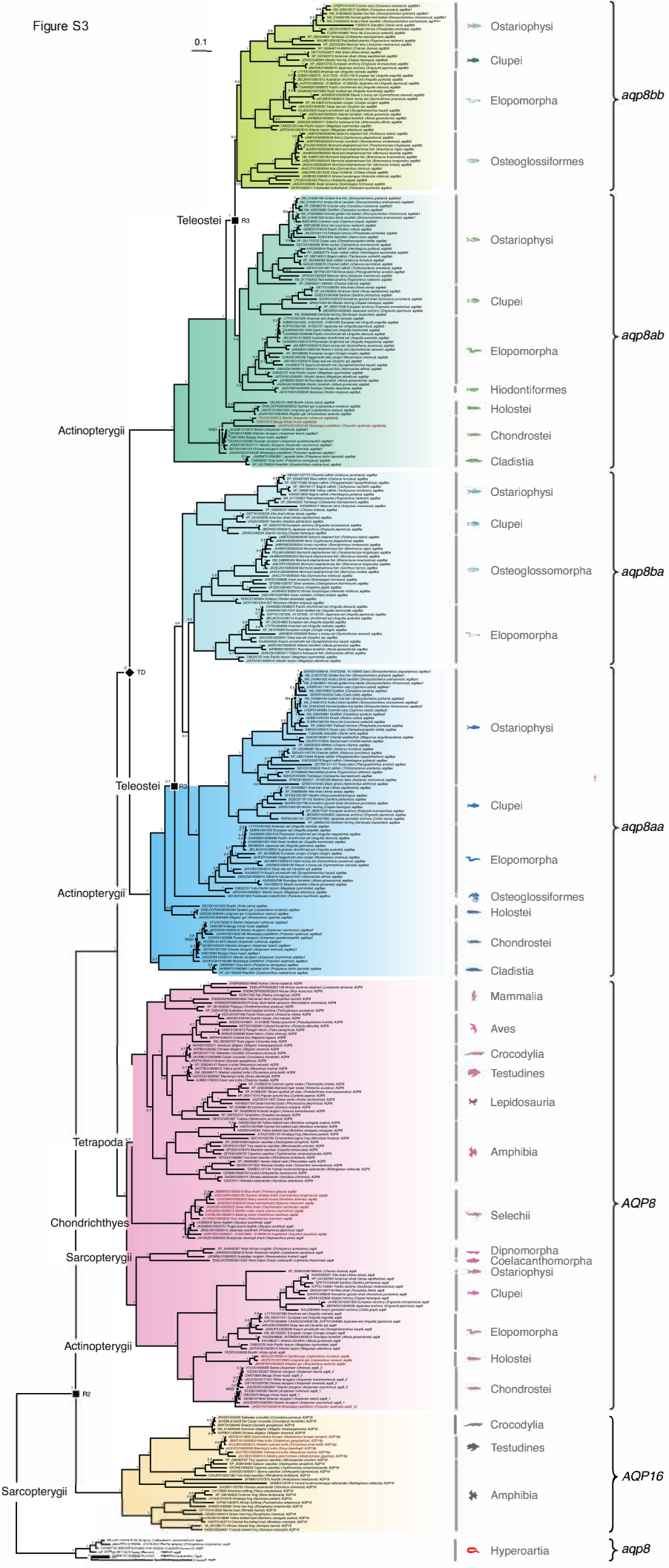

Figure S4

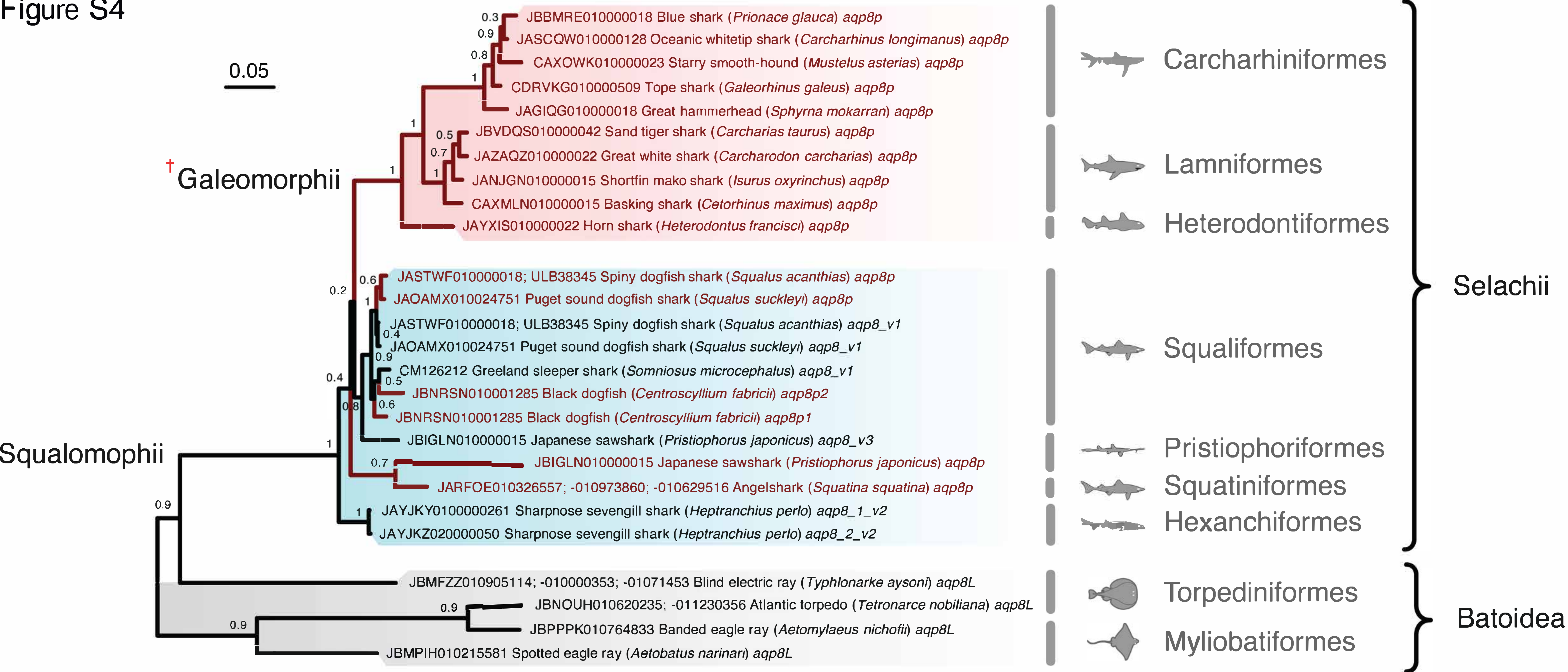

## Figure S5

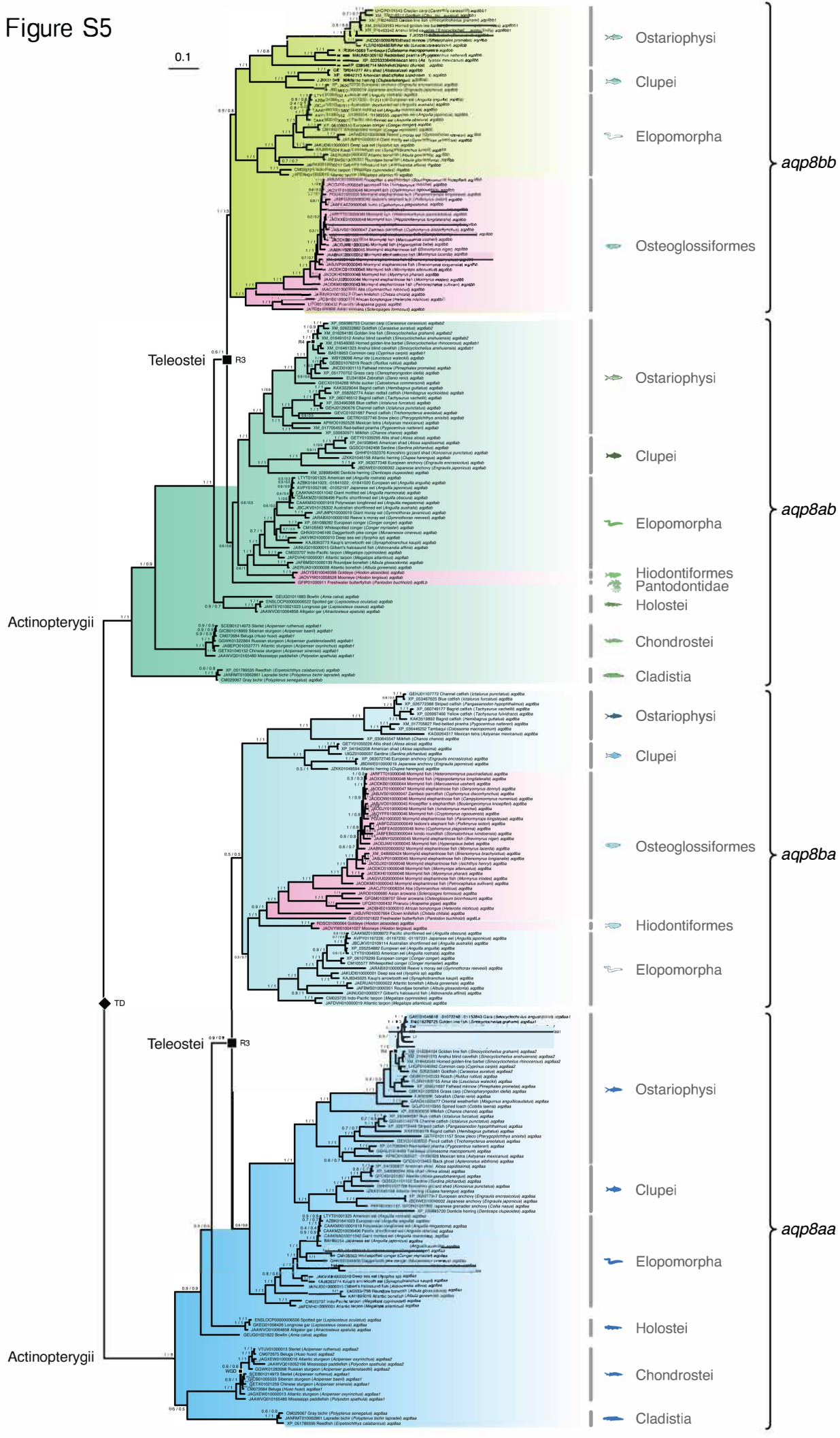

Figure S6

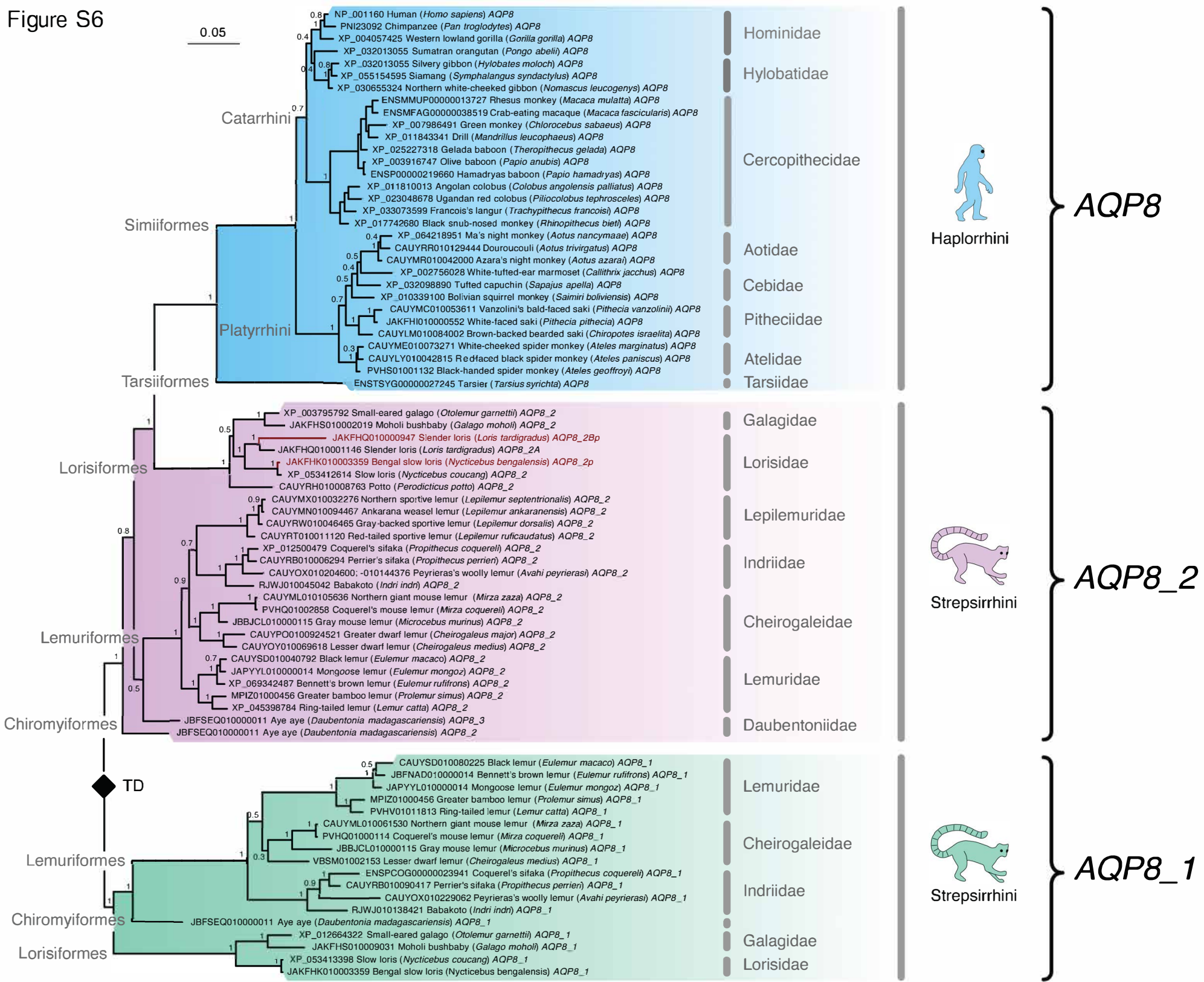

Supplement: Supplementary file 1 [file ijms-27-03937-s001.zip › ijms-4232752-supplementary.pdf]
